# Effect of Different Yeast Strains and Temperature of Fermentation on Basic Enological Parameters, Polyphenols and Volatile Compounds of Aurore White Wine

**DOI:** 10.3390/foods8120599

**Published:** 2019-11-20

**Authors:** Justyna Samoticha, Aneta Wojdyło, Joanna Chmielewska, Joanna Nofer

**Affiliations:** 1 Department of Fruit, Vegetable and Nutraceutical Plant Technology, Wrocław University of Environmental and Life Sciences, 37 Chełmońskiego Street, 51-630 Wrocław, Poland; 2 Department of Fermentation and Cereal Technology, Wrocław University of Environmental and Life Sciences, 37 Chełmońskiego Street, 51-630 Wrocław, Poland; 3 Department of Chemistry, 25 Norwida Street, 50-375 Wrocław, Poland

**Keywords:** hybrid, yeast, white wine, phenolic compounds, volatile compounds, antioxidant capacity, alcohol

## Abstract

The aim of this study was to investigate the content of phenolics by Ultra Performance Liquid Chromatography–Photodiode Array (UPLC–PDA), and volatile compounds by Gas Chromatography–Mass Spectroscopy (GC–MS), antioxidant capacity by 2,2′-azinobis-(3-ethylbenzothiazoline-6-sulfonic acid) radical cation (ABTS^•+^) and ferric-reducing antioxidant power (FRAP) assay, and color of Comission Internationale de l’Eclairage system (CIE) L*a*b* cv. Aurora white wine depending on fermentation conditions (a temperature of 12 °C vs. 20 °C and type of natural and commercial yeast (*Saccharomyces cerevisiae* vs. *Saccharomyces bayanus*)). The final wine differed in the content of total phenolic compounds (201.0–287.2 mg/L), except for the variants fermented at 20 °C with *S. cerevisiae* (321.9 and 329.4 mg/L for *S. cerevisiae* as Challenge Aroma White and SIHA^®^ Cryaroma type, respectively). A decrease in antioxidant activity ranging from 43.3% to 65.4% (ABTS and FRAP assay) in the matured wine vs. must was demonstrated. *S. cerevisiae* wine was also characterized by the highest content of total volatile compounds (3.7–4.2 mg/L vs. 1.3 mg/L in the must). In general, the wine obtained with *S. cerevisiae* had higher alcohol content, antioxidant capacity, and was richer in polyphenolic and volatile compounds.

## 1. Introduction

Wine is a product of fermentation, a process conducted by a population of microorganisms composed of many species of yeasts and bacteria. The addition of commercial yeast strains makes fermentation safer and easier to control. *Saccharomyces cerevisiae* is the most frequently chosen yeast. The dynamics of a spontaneous fermentation are often unpredictable and may introduce less desirable traits into wine that may even be spoiled. However, wild yeasts are capable of producing high-quality and unique-flavored wines [1]. During alcoholic fermentation, yeasts carry out the biotransformation of grape into wine compounds by converting sugars into ethanol and other metabolites [2].

In recent years in Poland, there has been a rapid expansion of grape growing, particularly for wine production. Wines produced in a cool climate may compete with those from traditional wine countries and they often reveal unique properties [3]. Due to climatic conditions, white wine production is preferred in Poland [4]. The climatic conditions vary greatly, and summers are longer, with higher temperatures (but not very hot) with increasing altitude and distance from the sea, and from east to west. This may affect the accumulation of flavonoid compounds in grapes. Therefore, in the past few decades, many viticultural researchers have focused on the effects of the environment in which the grapes are grown [5]. Cold climate wines are characterized by different chemical composition in terms of alcohol content and the quantity and type of aromatic compounds. Recently, alcohol beverages with reduced alcohol content (9–13%, *v*/*v*) have become more popular among consumers [6], but the content of ethanol is very important for wine quality. It may modify the sensory perception of aromatic attributes as well as the detection of volatile compounds. Through interactions with tannins and aromas, ethanol also affects wine viscosity, body, and perception of sweetness, sourness, bitterness, saltiness, astringency, aroma, and flavor [7,8]. Kourkoutas et al. [9] suggested that ethanol production at 5 °C was equal to production during traditional fermentation at 22–25 °C, but the concentrations of higher alcohols were lower and fruity aroma and fine taste improved the quality of wine fermented at a low temperature. Higher alcohols, i.e., secondary yeast metabolites, may have both positive and negative impacts on wine aroma and flavor. The use of different yeast strains during fermentation contributes to variable higher alcohol profiles and their concentrations in the final wine [10]. Summarizing the current literature data [11], the resulting modifications in wine volatile and non-volatile fractions could be a consequence of yeast activity and modifications of their metabolism and subsequently its by-products.

In recent years, inactive dry yeast preparations have gained popularity and have been used during the wine-making process to obtain a stable sensory evaluation through modulated aromatic composition which enhanced interest among winemakers [12]. Dry yeasts are also used as alcoholic fermentation enhancers, promoting yeast resistance to osmotic stress or improving nitrogen compound assimilation and counteracting the development of wild microflora [11].

Aurore, also known as Seibel 5279 or Feri Szoeloe, Financ Szoeloe, and Redei, is a white complex hybrid grape cultivar that ripens early between August and September. Despite the fact that Aurore is used to produce white wine, it often has a pink tinge, similar to Pinot gris or Gewürztraminer but is less intense. Aurore is used to produce a wide range of both blended and varietal white wine styles at a variety of sweetness from dry to off-dry, however less often sparkling wine. Wines obtained from cv. Aurore are light-bodied floral wines relatively neutral in flavor but often with a characteristic “foxy” note typical of many hybrids. Normally, cv. Aurore is planted when the season is short because it is winter-hardy, able to sustain winter frost down to even −29 °C, shows high resistance to downy mildew, powdery mildew, black rot and botrytis bunch rot. The cultivar is popular, e.g., in Canada, the United Kingdom or the USA, but its popularity is steadily growing in Poland and other European countries with a similar cold climate.

This and other white hybrid cultivars are the most widely planted throughout Poland, Slovenia, Slovakia, Czech Republic, Hungary, Romania and Ukraine. Considering the economic importance of wine-making in countries with a cold climate, it is necessary to find tools for improving the quality of their white wines by refining vinification protocols with respect to different yeasts or temperature of fermentation.

Therefore, the aim of this study was to compare the effects (i) of different inactive dry commercial yeasts (*Saccharomyces cerevisiae* and *Saccharomyces bayanus*) vs. native microorganisms present on the berries at different (ii) temperatures of fermentation on basic enological parameters, and additionally the content of polyphenols, antioxidants, and volatile compounds in Aurore white wine. Basic enological wine was assessed based on main parameters, such as ethanol and sugar content, pH titratable acidity, color parameters and aroma profiling. Additionally, the changes occurring in the must and during the fermentation and maturation processes were discussed. Finally, the content of polyphenolics as well as antioxidant capacity were determined. Results can be useful for winemakers, especially in Poland and other countries with similar climatic conditions.

## 2. Materials and Methods

### 2.1. Chemicals

Acetonitrile for chromatography analysis and ascorbic acid were purchased from Merck (Darmstadt, Germany). Formic acid, methanol, 2,2′-azinobis(3-ethylbenzothiazoline-6-sulfonic acid) (ABTS^•+^), 6-hydroxy-2,5,7,8-tetramethylchroman-2-carboxylic acid (Trolox), and 2,4,6-tri(2-pyridyl)striazine (TPTZ) were purchased from Sigma-Aldrich (Steinheim, Germany). Caftaric acid, (+)-catechin and procyanidin B2, quercetin-3-*O*-glucoside, myricetin-3-*O*-glucoside were purchased from Extrasynthese (Lyon, France). Divinylbenzene/carboxen/polydimethylsiloxane fiber ((DVB/CAR/PDMS), 50/30 μm, coating 2 cm) was purchased from Supelco (Bellafonte, PA, USA). K_2_S_2_O_5_, and NaOH were purchased from POCh (Gliwice, Poland). The yeast nutrient SIHA^®^ Proferm Plus nutrient, commercially yeast *S. cerevisiae* as SIHA^®^ Cryarome and *S. bayanus* as SIHA^®^ Active Yeast 4 were purchased from Eaton (Langenlonsheim, Germany). *S. cerevisiae* as Challenge Aroma White was purchased from Enartis (San Martino-Trecate, Italy).

### 2.2. Vinification Protocols

Aurore cv. was cultivated in Jaworek vineyard in Miękinia (51°10′30.11″ N, 16°45′6.87″ E), near Wrocław (Poland), with the typical climatological conditions of cold climate regions. Aurore grapes were harvested in 2017 at optimum technological maturity (density = 20.0° Bx; total acidity = 11.21 g/L; and pH, 3.24) and good sanitary conditions.

**Figure 1:**
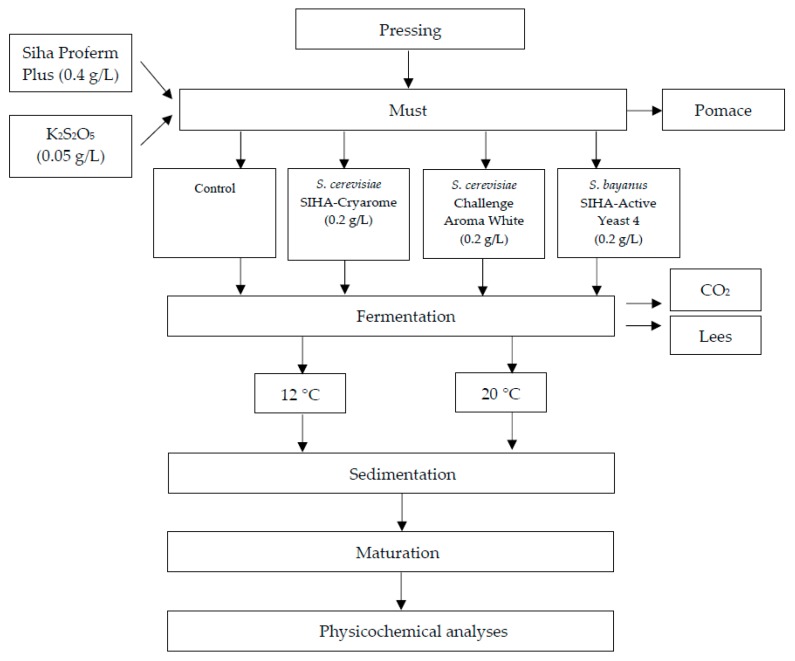
Scheme of the production of Aurore cv. white wine.

The white wine technology process is shown in Figure 1. Manually, harvest grapes (approximately 60 kg) were destemmed, crushed and pressed with a hydraulic press machine (TOYA, Wrocław, Poland).

Four types of Aurore wines were elaborated—in two replicates for each one (*n* = 2). For each vinification, 2.5 L of must was processed. The must was supplemented with yeast nutrient SIHA^®^ Proferm Plus nutrient (0.4 g/L), and bactericide was added as K_2_S_2_O_5_ (0.05 g/L). Fermentation was induced by yeast culture inoculation: *S. cerevisiae* as Challenge Aroma White, and SIHA^®^ Cryarome or *S. bayanus* as SIHA^®^ Active Yeast 4 at 0.2 g/L. The control sample was the must without inoculation by commercial yeast. The fermentation of Aurore must was performed at controlled temperatures: 12 and 20 °C. After the end of fermentation, the wines were racked in 2 L glass tanks and held for 4 months at 4 °C. The samples for chemical analyses were collected at every stage of the production (must, wine after fermentation (F) and after storage (W)).

### 2.3. Physicochemical Analyses

pH and titratable acidity (TA) were determined by titration aliquots (Schott Titroline 7500 KF Volumetric KF Titrator; Mainz, Germany) of 10 mL of must or wine, by 0.1 N NaOH to an end point of pH 7.0, using an automatic pH titration system and expressed as g of tartaric acid/L. Alcohol content in wine was determined by using an oscillating densimeter DMA 4500M (Anton Paar, Graz, Austria), with the result as expressed as the volume percent (% vol.). Sugar content was measured by HPLC, described previously by Samoticha et al. [13]. Results are expressed in grams per 100 mL. The color of must and wine was determined using an A5 Chroma-Meter (Minolta CR300, Osaka, Japan), referring to color space measured by Comission Internationale de l’Eclairage system as CIE L*a*b* where L*—brightness, a*—color from green (−a*) to red (+a*), and b*—color from blue (−b*) to yellow (+b*).

### 2.4. Analysis of Phenolic Compounds by Ultra Performance Liquid Chromatography–Photodiode Array (UPLC–PDA)

The samples were prepared as described by Wojdyło et al. [5]. Prior to analysis, must and wine samples were filtrated through a 0.22 μm membrane filter. The samples were analyzed by using an Acquity UPLC system (Waters, Milford, MA, USA) with a PDA detector (Waters, Manchester, UK). Chromatographic separations were performed on a UPLC BEH C18 column (1.7 µm, 2.1 mm × 100 mm; Waters Corporation, Milford, CT, USA) maintained at 30 °C. Elution was performed at a flow rate of 0.42 mL/min. The gradient started with 99% of 1% aqueous formic acid, isocratic conditions for 1 min, followed by a 11 min linear gradient of 1% to 40% acetonitrile with 1% formic acid applied. From 12 min, the acidified acetonitrile was increased to 100%, followed to 2 min, and then returned to initial conditions at 2 min. The volume injected was 5 µL. Polyphenolic compounds were subsequently tentatively identified by liquid Chromatography–Electrospray Ionization–Mass Spectrofotometer/Mass Spectrofotometer Quadrupole Time–of–Flight (LC–ESI–MS/MS QTof) as described previously by Wojdyło et al. [5] by comparing the areas and retention times with standards and considering the generated fragments and data from the literature. Calibration curves were made from selected standards: (−)-epicatechin, (+)-catechin, procyanidin B2 for flavan-3-ols evaluated at 280 nm, caftaric acid for phenolic acids at 320 nm, quercetin-3-*O*-glucoside, and myricetin-3-*O*-glucoside for flavonols at 360 nm. The sum of phenolic acids, flavan-3-ols and flavonols was estimated by summing the content of each compound identified by chromatography analysis, respectively. Quantification was achieved by injection of solutions of known concentrations ranging from 0.05 to 0.5 mg/mL (*R*^2^ ≤ 0.998). The results were expressed as mg/L for must and wine.

### 2.5. Determination of Antioxidant Capacity

The free radical scavenging capacities in must and wine were determined using the ABTS^•+^ method described by Re et al. [14] and the ferric-reducing antioxidant power (FRAP) method described by Benzie and Strain [15]. Determination methods were performed using a UV-2401 PC spectrophotometer (Shimadzu, Kyoto, Japan). All antioxidant capacity analyses were performed in triplicate, and results were expressed as micromoles of Trolox per 100 mL.

### 2.6. Analysis of Volatile Compound Measurement by Gas Chromatography–Mass Spectrometry (GC–MS)

Volatile compounds were analyzed using the headspace solid–phase microextraction (HS–SPME) technique described previously by Wen Chua et al. [16]. Extraction was performed using SPME fiber and analysis. The sample (5 mL) was placed in a 10 mL glass vial. The vial was sealed with a Teflon-faced septum cap, and the sample was pre-conditioned at 40 °C for 15 min. Microextraction lasted for 40 min at 40 °C with stirring (1100 rpm). For desorption, the fiber was inserted into the GC–MS injector port for 10 min (3 min in the splitless mode). The fiber was withdrawn into the needle and injected into the injection port of the GC–MS at a temperature of 220 °C for 3 min. GC–MS analysis was conducted on a Varian CP-3800/Saturn 2000 (Varian, Wallnut Creek, CA, USA) equipped with a ZB-5MS capillary column (30 m × 0.25 mm inner diameter (i.d.) × 0.25 μm film thickness). The initial oven temperature was kept at 50 °C, raised to 130 °C at a rate of 4 °C min^−1^, subsequently increased to 180 °C at 10 °C min^−1^, and finally increased to 280 °C at a rate of 2 °C min^−1^. Helium was used as a carrier gas at a flow rate of 1.0 mL min^−1^. The samples were manually injected in a split mode (1:10). Mass spectra were obtained in an electronic ionization (EI) mode of 70 eV, with a scan range of *m*/*z* 35–550. After each extraction was completed, the SPME fiber was placed in the injection port of GC–MS at 220 °C for 15 min to ensure that the volatiles were completely eluted to prepare for the subsequent extraction. A mixture of aliphatic hydrocarbons soluble in methanol in the range from C-5 to C-23 was used as a standard for determining retention indexes. Volatiles were identified by comparing the mass spectra of the compounds obtained experimentally, and the mass spectra were available in the National Institute of Standards and Technology (NIST 14) database. Identification of compounds is based on Kovats retention time indices, which allow you to assign a specific smell to a given compound.

### 2.7. Statistical Data Processing

Vinification for all sample was carried out in duplicate and all chemical analyses were performed in triplicate. The obtained data were processed and expressed as mean values and standard deviations. All statistical analyses were conducted using Statistica version 12.5 (StatSoft, Krakow, Poland). Significant differences (*p* ≤ 0.05) between means were evaluated by two-way ANOVA (yeast and temperature) and Duncan’s range test.

## 3. Results and Discussion

### 3.1. Ethanol pH, Total Acidity, Sugars Content and Color of Must and Wine

The type of yeast and temperature of fermentation did not significantly affect ethanol production, pH or total acidity (*p* > 0.05; Table 1). Ethanol content ranged from 10.8% to 12.2% *v*/*v*. However, in the case of spontaneous fermentation, ethanol content was lower in the wine fermented at 12 °C (10.8% *v*/*v*) than at 20 °C (11.8% *v*/*v*). Similar results were reported by Versari et al. [17] for red wines from grapes, with an alcohol content of 11.9%. According to literature [18], key matrix constituents and the relative proportion of water:ethanol in alcoholic beverages as well as their interactions with other matrix components (e.g., sugars, acids, and tannins) can also have significant effects on the behavior of volatile compounds responsible for aroma and flavor. Alcohol content determines the formation of esters and other carbonyl compounds necessary in wine-making. This implies that the concentration of ethanol affects the characteristic quality and flavor of the finished product.

Low total acidity and high pH values in our study probably facilitated the yeast metabolism to some extent and were responsible for a relatively high alcoholic strength reached in the wine with dry yeast vs. the control sample [18,19].

The pH of the fresh wine was from 3.0 to 3.1. Maturation further lowered the pH down to 2.8–2.9. The total acidity of the must from cv. Aurore was 11.1 g tartaric acid/L and decreased during the wine-making process. Fresh wines were characterized by acidity ranging from 8.7 g to 10.4 g tartaric acid/L (wines fermented at 12 °C) and from 8.9 g to 10.7 g tartaric acid/L (at 20 °C). No significant changes (*p* < 0.05) caused by temperature or yeast were perceived. Maturation contributed to a further decline in acidity, and significant differences were observed for different yeast strains (*p* < 0.05). The wine fermented without commercial yeast had the lowest acidity at both temperatures (mean 8.5 g tartaric acid/L), and it was the highest in the wine obtained with *S. cerevisiae* SIHA^®^ Cryarome (mean 10.0 g tartaric acid/L). In general, the acidity of wine from cool climate countries is higher.

The content of organic acids in the must and wine depends on grape variety, ripeness, climatic conditions during ripening, the grape processing method, conditions of alcohol fermentation and wine storage [20]. Wine acidity is responsible for its sensory characteristics and is important for its preservation. Wines with low pH and a high content of organic acid are less sensitive to microbial spoilage and show more stable coloration [21,22]. Dobrowolska-Iwanek et al. [3] investigated the acidity of wines from southern Poland and found the acidity of wines from cvs. Aurore, Bianca and Jutrzenka to be 3.1, 3.3, and 3.9 g of tartaric acid/L, respectively. Tarko et al. [4] examined cool-climate grapevine cultivars from Alden, Bianca, and Aurore and reported their acidity to be 9.8, 9.3, and 7.7 g of malic acid/kg, respectively. In our study, the acidity of Aurore wine was 6.8 g of malic acid/L.

During fermentation, 82–96% of the initial amount of sugar was processed into alcohol. The yeasts selected for this study showed a similar pattern of sugar utilization. Moreover, samples that had undergone spontaneous fermentation and with alcohol content at the lowest showed statistically higher residual sugars level (Table 1). No effects of the fermentation temperature were observed (*p* > 0.05), but the efficiency of sugar processing during fermentation depended on yeast strain (*p* < 0.05). Additionally, non-*Saccharomyces* yeasts, especially native ones, disappear because of their weaker ethanol tolerance and inability to survive at its increasing concentration.

The color of Aurore must and wine was measured in the CIE L*a*b* system involving three components: L*—brightness, a*—color from green to red, and b*—color from blue to yellow. A color of pomegranate for fermented beverage was an important parameter for consumer acceptance [23,24]. An important factor in wine-making is that some of the initial color of the must is modified during vinification. The must of Aurore cv. characterized by L* = 34.8, a* = −0.26 and b* = 1.9. L* did not change significantly (*p* > 0.05) for different yeast strains or the temperature of fermentation. Fresh wines were slightly darker (L* from 32.1 to 34.0), probably due to the retained presence of yeast. Parameter a* was not affected by the fermentation temperature but it significantly depended on the yeast strain (*p* < 0.05). In the wine fermented with *S. cerevisiae* SIHA^®^ Cryarome, a* increased to the highest degree that corresponded to a redder shade. Parameter b* that is responsible for yellow color did not change significantly (*p* > 0.05) in the presence of commercial yeast used in this study. However, significant changes (*p* < 0.05) were noticed in the fresh wine depending on the temperature of fermentation and the wine from the must fermented at 20 °C was less yellow (3.0) than at 12 °C (3.8). A point worth mentioning is that all variations in color data reported here occurred exclusively during the fermentation stage, the color remaining almost unchanged after that (*p* > 0.05). Among the main problems of white wine production is its browning. The brown color results from an increase in yellow pigments [24,25]. Despite that, Aurore wine tested in this study did not darken but was still of bright yellow color after five months of storage. The wine color visibly changed its tonality after maturation. These variations could be linked to phenolic compounds, i.e., flavonols, flavan-3-ols and phenolic acid degradation recorded in the initial period of wine elaboration.

### 3.2. Quantification of Phenolic Compounds in Must and Wine

Three groups of phenolic compounds (phenolic acids, flavan-3-ols and flavonols) were detected by UPLC–PDA analysis in Aurora cv. must and wine. The concentration of these compounds is very important in understanding the antioxidant potency of white wines.

The content of those chemicals, crucial for wine quality (Table 2), ranged from 207.8 to 287.2 mg/L in the must and from 201.0 to 329.4 mg/L in the final wine (variants fermented at 12 and 20 °C, respectively). Wine phenolic composition depends on the grapes from which they are partly extracted, and on the vinification conditions [3], including temperature, mixing, parameters of the fermentation vessel, pre-fermentative skin contact maceration, ethanol concentration, SO_2_, yeast strain, pH, and pectolytic enzymes [25]. Total phenolic compounds amounted to 312.5 mg/L in the fresh must. Fermentation triggered different changes in the content of phenolics but those caused by temperature were not significant (*p* > 0.05) in either fresh or matured wine. Contrary to that, the use of different yeast strains resulted in significant changes (*p* < 0.05) in the content of polyphenols. Both *S. cerevisiae* strains (SIHA^®^ Cryarome and Challenge Aroma White) produced wines richest in polyphenols. The wines fermented spontaneously and with *S. bayanus* were characterized by a lower content of total phenolics. Dobrowolska-Iwanek et al. [3] investigated white wines from different grape cultivars, grown in southern Poland, in which total phenolic content ranged from 280 to 510 mg of gallic acid/L. Aurore wine produced by fermentation with *S. cerevisiae* D 576 contained 300 mg of gallic acid/L. Tarko et al. [4] reported total polyphenol content in Aurore wine amounting to 463.0 mg of catechin/L. Red wine is known to be 10 times richer in polyphenolics and to have greater antioxidant capacity than white wine, and this variability is due to red wine grape must fermentation [26,27]. This is why white wine receives much less attention than red wine.

Flavan-3-ols were the major phenolics group in Aurore wine and they constituted almost 76% of total must phenolics. The must contained 236.4 mg of flavan-3-ols per L. Contrary to yeast strain, the temperature of fermentation did not induce significant (*p* > 0.05) changes in flavan-3-ol content. The lowest concentration of flavan-3-ols was detected in the wine obtained with *S. bayanus* (154.4 and 183.3 mg/L, respectively for fresh and aged wine), and the wine fermented spontaneously (162.5 and 198.2 mg/L). The wines produced with *S. cerevisiae* were characterized by the highest amount of flavan-3-ols. This was particularly visible for the SIHA^®^ Cryarome strain that yielded wine in which these compounds were present at 290.1 and 301.7 mg/L in the fresh and matured wine, respectively.

An increase in flavan-3-ols during maturation was noticed for all variants, as compared with the wine immediately after fermentation. Flavan-3-ols are important for wine properties. Catechins and procyanidins strongly affect the susceptibility of white wine to browning, as flavan-3-ols may undergo oxidation and polymerization. They also have a direct impact on the complexity of wine taste and mouthfeel and are responsible for wine bitterness and astringency [28,29,30]. Additionally, Ma et al. [31] reported higher levels of flavan-3-ol sulfonated in aged wines compared to native flavan-3-ol monomers in most of the samples of young wines. Ma et al. [31] postulated that these sulfonated flavanols arise from a cleavage of an interflacan bond of proanthocyanidins and nucleophilic attack by SO_2_ on C4 carbocation. These results demonstrate that the sulfonation of tannins readily occurs under wine aging conditions, releasing flavan-3-ol sulfonates in significant quantities. If the amounts of sulfonated dimers, trimers and larger oligomers are also greater than the native proanthocyanidins, such a large fraction of modified condensed tannin affects wine taste and astringency. These results are in agreement with the structure explained by Foo et al. [28], where sodium epicatechin-(4β)-sulfonate formed in a reaction of condensed tannin with bisulfite at pH 5.5 and 100 °C. Also, Mattivi et al. [32] demonstrated that epicatechin-(4β)-sulfonate and procyanidin B2-4β-sulfonate were generated in a reaction of apple tannins with bisulfite in a model wine.

Phenolic acids are responsible for taste aspects such as wine bitterness and astringency [30]. The must analyzed in this study contained the highest amount of phenolic acids (75.0 mg/L), and their concentration decreased along the wine-making process. Statistical differences (*p* < 0.05) were regarding the temperature factor. A higher fermentation temperature (20 °C) allowed for retaining more phenolic acids than at 12 °C in both fresh and matured wine. The average content of phenolic acids in the wine after fermentation was 36.8 and 12.8 mg/L at 20 and 12 °C, respectively. In the variants of wine fermented at 20 °C, the content of phenolic acids decreased significantly after maturation, while it remained at a similar level in the wine fermented at a lower temperature. Yeast strain had no effect on the content of phenolic acids in the wine after fermentation, but significant changes were detected following maturation (*p* < 0.05). Phenolic acid content was the lowest in the matured wine produced by spontaneous fermentation (12.5 mg/L), while it was two times higher (26.0 mg/L) in the wine fermented by *S. cerevisiae* Challenge Aroma White. Some recent research demonstrate that the antioxidant capacity of white wine is much more associated with the presence of hydroxy cinnamates than with any other class of wine constituents.

Cinnamic acid derivatives represent the major group of white wine phenols and possess remarkable antioxidant capacity [33,34]. Flavonols are yellow pigments responsible for the color of white wine [13]. White wines are usually made from free running juices, without grape mash, having no contact with the grape skins. For this reason, flavonols were the least common phenolics in Aurore must and wine. The initial content of these compounds in the must was 1.1 mg/L, and it decreased in all tested variants during Aurore wine production. The final wine contained 0.4 to 1.0 mg/L of flavonols. Statistical analysis revealed no significant changes (*p* > 0.05) depending on the fermentation temperature or yeast strain. Flavonols are located in the peel of the grapes and their higher concentrations are observed in red wines as they are subjected to the process of peel maceration. The concentration of flavonols in white wine increases during skin fermentation and subsequently begins to decrease as phenols bind with proteins and yeast hulls (cell remnants), and precipitate. During fining, maturation and aging, the concentration of phenolic compounds continues to decrease [26]. Therefore, some modifications of white must maceration are being implemented to increase the content of polyphenolic compounds, especially flavonols in white grape must.

### 3.3. Antioxidant Capacity of Must and Wine

**Figure 2:**
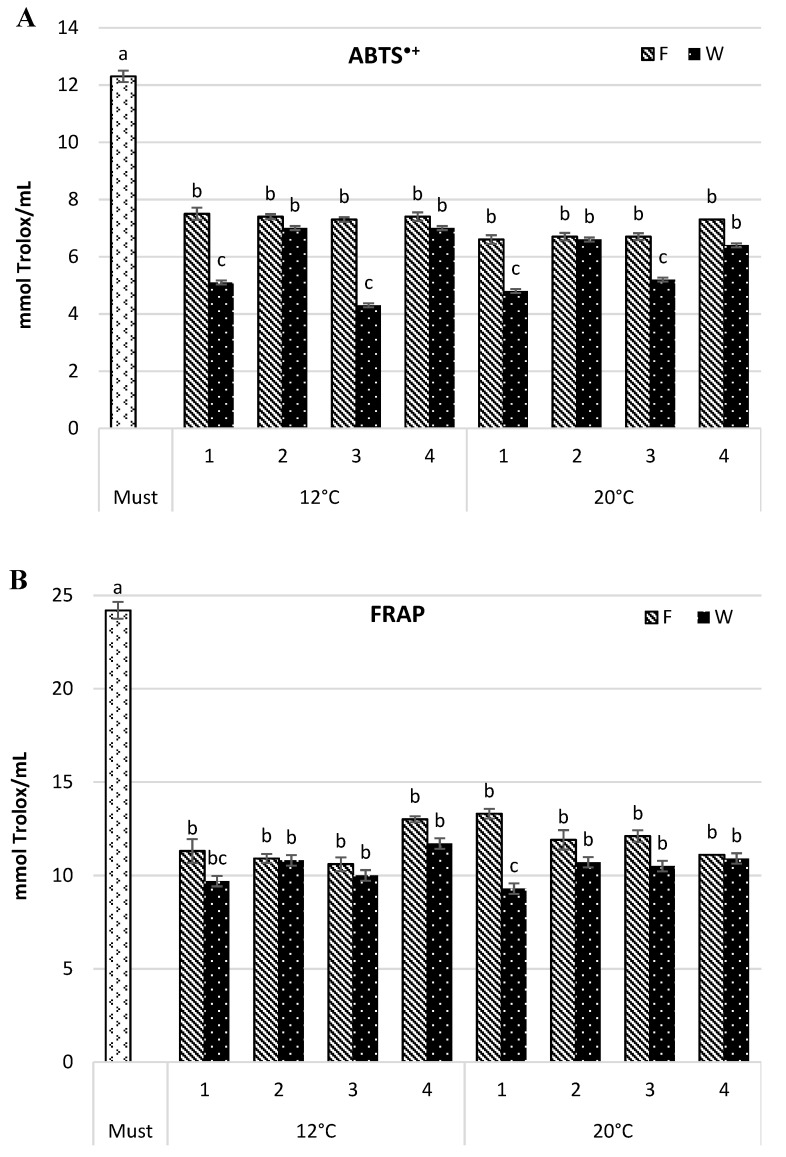
Antioxidant capacity (mmol Trolox/mL) by 2,2′-azinobis-(3-ethylbenzothiazoline-6-sulfonic acid)] radical cation (ABTS^•+^) (**A**) and ferric-reducing antioxidant power (FRAP) (**B**) in Aurore must and wine. F—wine after fermentation; W—wine after maturation. Yeast strain: 1—Spontaneous fermentation, 2—*S. cerevisiae* SIHA^®^ Cryarome, 3—*S. cerevisiae* Challenge Aroma White, 4—*S. bayanus* SIHA^®^ Active Yeast 4. Values (a, b, c) followed by the same letter were significantly different (*p* < 0.05) according to Duncan’s test.

The antioxidant capacity of wine samples was estimated by their ability to scavenge the ABTS^•+^ radical capacity and ferric-reducing antioxidant potential (FRAP) assays. Both assays confirmed the highest antioxidant capacity in the must (12.3 and 24.2 mmol Trolox/mL for ABTS^•+^ and FRAP, respectively) (Figure 2), and its reduction along consecutive steps of the wine-making process. The changes in intensity depended on the fermentation temperature and yeast strain. The maturation step also affected the antioxidant capacity of the tested wine.

In the wine fermented at 12 °C, the antioxidant capacity measured by ABTS^•+^ was reduced, as compared with the must, from 39.1% to 40.7% in the young wine, and from 43.1% to 65.1% following maturation. In the wines fermented at a higher temperature (20 °C), the decrease in antioxidant capacity in the fresh wine was greater and ranged from 40.7% to 46.4%, but the change after maturation was smaller and amounted to 46.4–60.8%. Differences induced by yeast strain were similar for both temperature variants and they were more pronounced in the wine after maturation. The wines obtained by spontaneous fermentation and by using *S. cerevisiae* Challenge Aroma White had the lowest antioxidant capacity. It amounted to 5.1 and 4.3 mmol Trolox/mL for 12 °C wines and 4.8 and 5.2 mmol Trolox/mL for 20 °C wines, respectively.

The results yielded by the FRAP assay showed a comparable trend. A similar level of reduction in antioxidant capacity, not significant, was noticed for both temperature variants (46.2–56.2% and 51.7–60.0% for 12 °C, and 45.1–54.2% and 55.0–61.6% for 20 °C, for fresh and matured wine respectively). The assay also confirmed that spontaneous fermentation and *S. cerevisiae* Challenge Aroma White strain most effectively reduced wine antioxidant capacity than the remaining samples (*p* ≤ 0.05). *S. cerevisiae* SIHA^®^ Cryarome and *S. bayanus* SIHA^®^ Active Yeast 4 produced wines with the highest antioxidant capacity, regardless of the fermentation temperature. Dobrowolska-Iwanek et al. [3] measured antioxidant capacity by FRAP as mM Fe/L and compared wines from a few white cultivars. In cv. Aurore wine, the antioxidant capacity amounted to 1.01 mM Fe/L and was lower than in cvs. Jutrzenka and Muscat Odeskij but higher than in the wines from cvs. Bianca or Sibera. Tarko et al. [4] compared two Polish wines, white Aurore and red Rondo, and found that the antioxidant capacity (ABTS^•+^) was approximately eight times lower in the white wine and amounted to 1.1 mg of Trolox/L (4.4 mmol Trolox/mL). Our results were concurrent with those referenced above.

As per literature [3,25], different wines have different quantities and spectra of native antioxidants and therefore different health benefits. Wine antioxidant capacity markedly depend on the content of phenolic compounds and other factors such as grape cultivar, soil, nutrition, climatic conditions, weather, wine-making procedure, and conditions of maturation and storage [25].

The potential health effects of moderate wine consumption have also been linked to wine flavonoid content [33]. Whereas nonflavonoid phenolics are almost equally distributed in white and red wine, the flavonoid content differs significantly. In white wine, flavonoids commonly constitute up to approximately 20% of the total polyphenol content, whereas in red wines, flavonoids account for over 85% of the phenolic substances. As flavonoids originate from the skins, seeds, and stems of grapes, their extraction yield during vinification is mainly influenced by factors such as temperature and length of skin contact. Therefore, the flavonoid content in red wine is approximately 20–30 times higher (>1000 mg/L) than in white wine [34,35]. Some literature data [30,33] indicate that white wines are rich in polyphenols and have antioxidant properties similar to those of red wines and can therefore also help to prevent arteriosclerosis and coronary heart diseases. The antioxidant properties of the phenolic compounds have been widely studied but also the prooxidant effects of white wine on low-density-lipoprotein (LDL) oxidation have been reported [36]. Other investigators [37] found that white wine polyphenols were more effective than red wine polyphenols in the in vitro inhibition of LDL oxidation. For white wine polyphenols, the average Inhibitory Concentration (IC50) reached 1.7 μM, whereas it was 2.9 μM for red wines [37]. This finding was explained by differences in the polyphenolic composition of red and white wine, i.e., the predominance of polymeric phenols (tannins) in red wine and low-molecular-weight polyphenols in white wine. Additionally, some other factors are important, such as the absorption of a compound into blood circulation, metabolism and excretion, as these processes determine the health-promoting effects of polyphenols. It was often demonstrated that food-derived low-molecular-weight polyphenolic compounds are more beneficial due to their greater bioavailability and absorption. Current knowledge indicates that proanthocyanidin dimers and trimers are absorbed in their intact form and their absorption rates are less than 10% of (−)-epicatechin [38].

### 3.4. Content of Volatile Compounds in Must and Wine

Aroma is an important factor determining the quality of all foods, but in the case of wine, it is probably among the most important indicators contributing to consumer attraction and enjoyment. A distinctive bouquet and flavor of wine depends on several factors such as the type of cultivar, year of harvest, fruit ripening, wine fermentation, maturation of the final product, and production method [39]. Aurore wine and must confirmed the presence of 52 volatile compounds that belonged to different chemical families, including fatty acids, alcohols, esters, phenols, C_13_-norisoprenoids, monoterpenoids, and acetates (short-, medium- and long-chain ethyl and methyl esters). In this study and in the papers published by other authors [40], ten compounds, i.e., ethyl octanoate, ethyl decanoate, diethyl succinate, ethyl hexanoate, phenethyl acetate, 3-methylbutyl acetate, 3-methyl-1-butanol, 1-hexanol, 2-methyl-1-propanol, and 2-phenylethanol were identified as the main volatiles for the white wine aroma of Aurora cv. Table 3 shows the quantification of aroma constituents identified in Aurore wine. Total volatile compounds in the must amounted to 1.3 mg/L. In the course of the wine-making process, their content initially increased, particularly during fermentation (1.6–8.0 mg/L), but then decreased during maturation (1.6–4.2 mg/L). Esters constitute the most common chemical group in the volatile fraction of Aurore wines and musts and are associated with fruity and favorable properties in all types of wine. Ester content in the must amounted to 1.0 mg/L but the wine-making process contributed to its increase. Wine esters come from two distinct sources, i.e., enzyme esterification during fermentation and chemical esterification during long-term aging. The most prevalent wine esters include acetates of higher alcohols (formed in a reaction of acetyl-CoA with higher alcohols) and ethyl esters of fatty acids (formed by esterification of fatty acids and ethanol). Contrary to yeast strain, fermentation temperature did not significantly affect (*p* > 0.05) ester content. Fresh wine produced by *S. cerevisiae* SIHA^®^ Cryaroma featured the highest (6.5 mg/L), and the wine fermented spontaneously the lowest (1.4 mg/L) number of esters. The effect of maturation period and different fermentation temperature induced several changes in the aroma profile of Aurore wines. As expected, the number of esters decreased after storage but was still higher than in the must. The same non-significant role of the fermentation temperature was confirmed for the matured wine.

*S. cerevisiae* strains yielded wines with the highest content of esters (3.7 and 3.4 mg/L for SIHA^®^ Cryarome and Challenge Aroma White, respectively), as compared with other yeast strains. With regard to storage time, a significant decrease in the concentration of short-chain fatty acid ethyl esters and ethyl acetates was reported. This was concurrent with the results published by Palomo et al. [40] and Cejudo-Bastante et al. [39], who studied the aroma and compositional changes in the wine under storage conditions. However, the levels of other ethyl and methyl esters such as heptadecanoate, hexadecanoate and pentadecanoate increased during storage. Ethyl octanoate, ethyl decanoate and ethyl dodecanoate were found at the highest concentrations in Aurore white wine. Fermentation temperature is among the strongest indicators of the ester type formed [41,42]. However, the concentration of volatile compounds followed a very similar pattern: low fermentation temperature induced a rise in the proportion of all esters and fatty acids but reduced the total concentration of volatile alcohols. This may be due to the higher production and retention of esters during fermentation at a lower temperature [43]. The results published by Molina et al. [43] and Beltran et al. [44] also suggested that a lower fermentation temperature (~15 °C) was optimal for the effective production of the key esters during wine-making. Esters determine the fruity aroma of wines [45]. The main esters are fatty acid ethyl esters and acetates such as ethyl acetate, ethyl butyrate, ethyl hexanoate, ethyl octanoate, ethyl decanoate, hexyl acetate, isoamyl acetate, isobutyl acetate, and phenylethyl acetate [46].

The second major group of volatile components in Aurore wine comprised alcohols that are produced during sugar catabolism or decarboxylation and deamination of amino acids by yeast during fermentation. Compounds Such as 3-methyl-1-butanol and 2-phenylethanol were the most abundant constituents among all identified alcohols. According to the literature [41,43,44,46] on the volatile composition of several types of white wines analyzed by solid–phase microextraction followed by gas chromatography–mass spectrometry method (SPME–GC–MS), 3-methyl-1-butanol and 2-phenylethanol were described as responsible for the characteristic fruity and rose odor of wine. In this work, the concentration of all alcohols significantly increased during the storage period. A similar effect was reported by Garde Cerdan et al. [47]. Moreover, different fermentation temperature also induced changes in Aurore wine volatiles. A comparison of two major volatile alcohols at different fermentation temperatures showed a significant increase in their content when the must fermentation was carried out at 20 °C vs. 12 °C. However, the wines fermented at a lower temperature featured a higher content of amyl alcohols. Beltran et al. [44] and Yilmaztekin et al. [48] reported an increase in the concentration of all higher alcohols with increasing fermentation temperature, whereas Molina et al. [43] showed that only the concentration of 2-phenylethanol increased in this situation. Some work reported that a level of higher alcohols below 300 mg/L confers a desirable complexity to the wine, whereas concentrations over 400 mg/L can have a negative effect [49].

With regard to other compounds, the number of terpenes, ketones and aldehydes did not show significant variations depending on the time of storage and different fermentation temperature; only a slight decrease in the concentration of β-demascenone and citronellol was observed. These compounds are responsible for the violet and floral note, and fruity-flowery and citrus notes, respectively. Similar results were obtained by Zhang et al. [50].

In general, raising the fermentation temperature from 12 to 20 °C significantly increased the concentration of almost all volatile esters and fatty acids, contrary to the content of alcohols. Torija et al. [51] demonstrated that the content of esters and acetates decreased as a consequence of raising the fermentation temperature from 13 to 25 °C, which may markedly affect the aroma of white wine.

In analyzed Aurore wine, the content of methyl anthranilate and 2-aminoacetophenone is well below the thresholds (1 ug/L; data not present). These compounds, key odorants responsible for “foxiness” and “American grape aroma”, are typically for *Vitis rotundifolia* or *Vitis lambrusca* grapes, juices or wines. However, they are undetectable in *Vitis riparia* and *Vitis cinereal* wines [52]. According to Robinson et al. [53] Aurore wine are relatively neutral in flavor but often exhibit the characteristic foxy note typical of many hybrids. Nevertheless, some author [54] observed that this foxy aroma tends to be more pronounced in wines produced in the eastern United States than in the west. The formation of 2-aminoacetophenone in grape is favored by several factors, such as reduced nitrogen fertilization combined with hot, dry summers, and the risk increases in wines produced with early harvested grapes. The key role of these compounds in ripening grapes may serve as a deterrent to birds. This odor is differently described as furniture polish, damp cloth, acacia blossom, naphthalene, and fusel alcohol, and causes a considerable number of wine rejections.

## 4. Conclusions

Wine is a sensitive and complex combination of chemical components that is shaped by the grape cultivar, conditions and location of cultivation, and the processes of production and storage. Alcohol content, organic acid, sugars, polyphenols and some aroma compounds (such as ethyl esters terpenols, acetates, or norisoprenoids) are considered to be character impact compounds for Aurore wine. This paper discussed the effects of fermentation temperature and different strains of yeast on Aurore wine composition. Different commercial yeast strains changed the chemical composition of wine. *S. cerevisiae* SIHA^®^ Cryarome and Challenge Aroma White yielded Aurore wine with the highest content of ethanol (12.0%–12.2%) and total polyphenols (283.1–308.3 mg/L) and a high antioxidant capacity (4.3–7.0 mmol Trolox/mL). This wine was also characterized by the highest content of total volatile compounds (3.7–4.2 vs. 1.3 mg/L in the must), as compared with the other samples.

## Figures and Tables

**Table 1:** Basic parameters of Aurore wine: ethanol content, pH, total acidity, sugar content, and color by CIE L*a*b.

Fermentation Temperature (°C)	Yeast	Ethanol (% *v*/*v*)	pH		Total Acidity (g Tartaric Acid/L)		Sugar (g/100 mL)		Color					
									L*		a*		b*	
Must		-	3.2		11.1		19.9		34.8		−0.26		1.9	
		F	F	W	F	W	F	W	F	W	F	W	F	W
12	Spontaneous fermentation	10.8 ± 0.8	3.1 ± 0.1	2.9 ± 0.1	10.4 ± 0.2	8.3 ± 0.4	3.6 ± 0.3	2.3 ± 0.7	32.9 ± 1.1	35.5 ± 1.1	−0.13 ± 0.0	−0.52 ± 0.0	3.7 ± 0.2	3.8 ± 0.3
	*S. cerevisiae* SIHA^®^ Cryarome	12.0 ± 0.6	3.1 ± 0.3	2.9 ± 0.2	10.1 ± 0.5	10.0 ± 0.6	1.0 ± 0.4	1.0 ± 0.1	33.8 ± 1.7	34.8 ± 1.9	0.43 ± 0.1	−0.09 ± 0.1	3.9 ± 0.2	4.7 ± 0.2
	*S. cerevisiae* Challenge Aroma White	12.2 ± 0.7	3.1 ± 0.2	2.9 ± 0.0	9.1 ± 0.5	9.1 ± 0.1	0.8 ± 0.1	0.8 ± 0.0	34.0 ± 0.9	35.8 ± 1.0	−0.13 ± 0.0	−0.56 ± 0.1	3.8 ± 0.1	3.9 ± 0.3
	*S. bayanus* SIHA^®^ Active Yeast 4	11.9 ± 0.8	3.1 ± 0.1	2.9 ± 0.1	8.7 ± 0.4	9.1 ± 0.2	1.5 ± 0.4	1.5 ± 0.2	34.0 ± 1.3	35.5 ± 1.5	−0.21 ± 0.1	−0.49 ± 0.0	3.8 ± 0.1	4.1 ± 0.4
20	Spontaneous fermentation	11.8 ± 0.9	3.0 ± 0.2	2.8 ± 0.1	8.9 ± 0.3	8.7 ± 0.1	2.2 ± 0.1	1.5 ± 0.3	32.6 ± 1.0	35.5 ± 1.4	−0.16 ± 0.1	−0.40 ± 0.0	3.6 ± 0.0	4.4 ± 0.4
	*S. cerevisiae* SIHA^®^ Cryarome	11.9 ± 1.0	3.0 ± 0.1	2.8 ± 0.0	10.7 ± 0.5	10.1 ± 0.4	1.1 ± 0.2	1.1 ± 0.2	32.1 ± 2.0	36.1 ± 1.5	−0.09 ± 0.0	−0.52 ± 0.0	2.8 ± 0.1	3.0 ± 0.2
	*S. cerevisiae* Challenge Aroma White	12.2 ± 1.1	3.1 ± 0.1	2.8 ± 0.2	9.6 ± 0.5	9.6 ± 0.9	1.3 ± 0.1	1.0 ± 0.1	32.5 ± 0.7	36.1 ± 1.7	−0.15 ± 0.0	−0.52 ± 0.1	2.6 ± 0.1	2.9 ± 0.5
	*S. bayanus* SIHA^®^ Active Yeast 4	11.9 ± 0.9	3.1 ± 0.1	2.9 ± 0.2	9.6 ± 0.4	9.4 ± 1.1	1.5 ± 0.1	1.5 ± 0.2	34.0 ± 1.2	35.8 ± 1.9	−0.21 ± 0.0	−0.41 ± 0.0	3.0 ± 0.2	3.0 ± 0.3
Yeast	Spontaneous fermentation	11.3 a	3.0 a	2.9 a	9.7 a	8.5 b	2.9 a	1.9 a	32.8 a	35.5 a	−0.15 b	−0.46 ab	3.6 a	4.1 a
	*S. cerevisiae* SIHA^®^ Cryarome	12.0 a	3.0 a	2.8 a	10.4 a	10.0 a	1.0 b	1.1 c	33.0 a	35.4 a	0.17 a	−0.31 a	3.4 a	3.9 a
	*S. cerevisiae* Challenge Aroma White	12.2 a	3.1 a	2.9 a	9.4 a	9.3 ab	1.1 b	0.9 c	33.2 a	35.9 a	−0.14 b	−0.54 b	3.2 a	3.4 a
	*S. bayanus* SIHA^®^ Active Yeast 4	11.9 a	3.1 a	2.9 a	9.1 a	9.2 ab	1.5 b	1.5 b	34.0 a	35.6 a	−0.21 b	−0.45 ab	3.4 a	3.6 a
Temperature	12 °C	11.7 a	3.1 a	2.9 a	9.6 a	9.1 a	1.7 a	1.4 a	33.7 a	35.4 a	−0.01 a	−0.42 a	3.8 a	4.1 a
	20 °C	11.9 a	3.0 a	2.8 a	9.7 a	9.4 a	1.5 a	1.3 a	32.8 a	35.9 a	−0.15 a	−0.46 a	3.0 b	3.3 a

F—wine after fermentation; W—wine after maturation. Values followed by the same letter, within the same column, were significantly different (*p* < 0.05) according to Duncan’s test; L*—brightness, a*—color from green to red, and b*—color from blue to yellow.

**Table 2:** Content of phenolic compounds in Aurore must and wine (mg/L).

Fermentation Temperature (°C)	Yeast	Phenolic Acids		Flavonols		Flavan-3-Ols		Ʃ Phenolic Compounds	
Must	-	75.0		1.1		236.4		312.5	
12		F	W	F	W	F	W	F	W
	Spontaneous fermentation	9.7 ± 1.1	10.6 ± 1.4	0.5 ± 0.1	0.6 ± 0.3	165.6 ± 3.6	210.3 ± 2.3	175.8 ± 5.2	221.6 ± 4.8
	*S. cerevisiae* SIHA^®^ Cryarome	10.7 ± 1.2	10.7 ± 1.8	0.3 ± 0.0	0.6 ± 0.1	248.4 ± 4.9	275.9 ± 1.7	259.3 ± 6.7	287.2 ± 3.9
	*S. cerevisiae* Challenge Aroma White	14.2 ± 0.9	14.8 ± 2.3	0.3 ± 0.0	0.8 ± 0.2	212.1 ± 5.2	228.7 ± 1.8	226.6 ± 4.4	244.3 ± 5.9
	*S. bayanus* SIHA^®^ Active Yeast 4	16.6 ± 2.1	15.6 ± 2.1	0.4 ± 0.0	1.0 ± 0.2	126.3 ± 2.9	191.2 ± 2.6	143.3 ± 5.9	207.8 ± 6.2
20	Spontaneous fermentation	23.0 ± 1.2	14.4 ± 1.7	0.2 ± 0.0	0.6 ± 0.1	159.5 ± 1.9	186.1 ± 2.8	182.7 ± 4.8	201.0 ± 6.2
	*S. cerevisiae* SIHA^®^ Cryarome	38.7 ± 2.5	24.6 ± 2.8	0.4 ± 0.1	0.6 ± 0.1	304.2 ± 2.4	355.1 ± 1.6	394.2 ± 5.7	329.4 ± 6.4
	*S. cerevisiae* Challenge Aroma White	40.0 ± 0.9	37.2 ± 2.0	0.2 ± 0.0	0.6 ± 0.0	261.1 ± 1.7	284.0 ± 1.5	301.3 ± 4.6	321.9 ± 7.4
	*S. bayanus* SIHA^®^ Active Yeast 4	45.6 ± 3.5	25.1 ± 1.9	0.3 ± 0.0	0.4 ± 0.0	182.5 ± 1.0	175.5 ± 1.3	228.4 ± 7.4	201.0 ± 3.9
Yeast	Spontaneous fermentation	16.4 a	12.5 b	0.4 a	0.6 a	162.5 c	198.2 b	179.3 b	211.3 b
	*S. cerevisiae* SIHA^®^ Cryarome	24.7 a	17.7 ab	0.3 a	0.6 a	290.1 a	301.7 a	326.8 a	308.3 a
	*S. cerevisiae* Challenge Aroma White	27.1 a	26.0 a	0.3 a	0.7 a	236.6 b	256.4 ab	263.9 a	283.1 a
	*S. bayanus* SIHA^®^ Active Yeast 4	31.1 a	20.3 ab	0.4 a	0.7 a	154.4 c	183.3 b	185.9 b	204.4 b
Temperature	12 °C	12.8 b	12.9 b	0.4 a	0.8 a	188.1 a	226.5 a	201.3 a	240.2 a
	20 °C	36.8 a	25.4 a	0.3 a	0.6 a	239.5 a	237.4 a	276.6 a	263.3 a

F—wine after fermentation; W—wine after maturation. Values followed by the same letter, within the same column, were significantly different (*p* < 0.05) according to Duncan’s test.

**Table 3:** Aroma of Aurore wine and must as a group of volatile compounds (mg/L).

Temperature of Fermentation (°C)	Yeast	Volatile Compounds													
		Esters		Alcohols		Aldehydes		Ketones		Terpenes		Other		Total	
Must	-	1.0		0.14		0.001		0.002		0.005		0.07		1.3	
		F	W	F	W	F	W	F	W	F	W	F	W	F	W
12	Spontaneous fermentation	1.4	1.4	0.13	0.16	0.001	0.0002	0.004	0.003	0.005	0.003	0.09	0.07	1.7	1.6
	*S. cerevisiae* SIHA^®^ Cryarome	7.6	3.9	0.19	0.27	0.004	0.003	0.005	0.004	0.009	0.005	0.15	0.10	8.0	4.2
	*S. cerevisiae* Challenge Aroma White	4.5	3.4	0.12	0.19	0.002	0.002	0.004	0.004	0.007	0.004	0.26	0.18	4.9	3.7
	*S. bayanus* SIHA^®^ Active Yeast 4	5.8	2.9	0.11	0.15	0.003	0.002	0.004	0.004	0.003	0.003	0.16	0.12	6.1	3.2
20	Spontaneous fermentation	1.4	2.4	0.14	0.17	0.001	0.001	0.002	0.002	0.007	0.004	0.10	0.06	1.6	2.6
	*S. cerevisiae* SIHA^®^ Cryarome	5.3	3.5	0.20	0.29	0.004	0.003	0.003	0.002	0.004	0.003	0.09	0.07	5.6	3.9
	*S. cerevisiae* Challenge Aroma White	5.1	3.4	0.21	0.26	0.006	0.004	0.003	0.002	0.01	0.005	0.23	0.14	5.6	3.8
	*S. bayanus* SIHA^®^ Active Yeast 4	6.0	2.6	0.12	0.18	0.003	0.002	0.004	0.003	0.008	0.003	0.15	0.10	6.3	2.9
Yeast	Spontaneous fermentation	1.4 c	1.9 c	0.13 bc	0.17 c	0.001 b	0.0006	0.003 a	0.002 a	0.006 ab	0.004 a	0.09 c	0.07 c	1.6 c	2.1 c
	*S. cerevisiae* SIHA^®^ Cryarome	6.5 a	3.7 a	0.20 a	0.28 a	0.004 a	0.003 ab	0.004 a	0.003 a	0.006 ab	0.004 a	0.12 c	0.09 c	6.8 a	4.1 a
	*S. cerevisiae* Challenge Aroma White	4.8 b	3.4 a	0.16 ab	0.22 b	0.004 a	0.003 a	0.004 a	0.003 a	0.008 a	0.005 a	0.25 a	0.16 a	5.2 b	3.8 a
	*S. bayanus* SIHA^®^ Active Yeast 4	5.9 a	2.7 b	0.11 c	0.16 c	0.003 a	0.002 b	0.004 a	0.003 a	0.005 b	0.003 b	0.16 b	0.11 b	6.2 ab	3.0 b
Temperature	12 °C	4.8 a	2.9 a	0.14 a	0.19 a	0.003 a	0.002 a	0.004 a	0.004 a	0.006 a	0.004 a	0.17 a	0.12 a	5.2 a	3.2 a
	20 °C	4.4 a	3.0 a	0.17 a	0.22 a	0.004 a	0.003 a	0.003 b	0.002 b	0.007 a	0.004 a	0.14 a	0.09 a	4.8 a	3.3 a

F—wine after fermentation; W—wine after maturation. Values followed by the same letter, within the same column, were significantly different (*p* < 0.05) according to Duncan’s test.
